# Impact of Baseline β-Catenin Comutations on Prognosis in EGFR-Mutant Lung Cancer

**DOI:** 10.1200/PO-24-00771

**Published:** 2025-08-06

**Authors:** Jonas Kulhavy, Katja Maurus, Miriam Blasi, Stephanie Brändlein, Simone Reu-Hofer, Julia Doll, Julia Böck, Albrecht Stenzinger, Daniel Kazdal, Jan Budczies, Valeria Roll, Volker Kunzmann, Elena Gerhard-Hartmann, Andreas Rosenwald, Ralf Bargou, Maria-Elisabeth Goebeler, Jens Kern, Pius Jung, Markus Krebs, Manik Chatterjee, Petros Christopoulos, Vivek Venkataramani, Horst-Dieter Hummel

**Affiliations:** ^1^ Comprehensive Cancer Center Mainfranken, University Hospital Würzburg, Bavarian Cancer Research Center (BZKF), National Center for Tumor Diseases (NCT) WERA, Würzburg, Germany; ^2^ Institute of Pathology, University of Würzburg, Würzburg, Germany; ^3^ Department of Thoracic Oncology, Thoraxklinik, Heidelberg University Hospital and National Center for Tumor Diseases (NCT), NCT Heidelberg, a Partnership Between DKFZ and Heidelberg University Hospital, Heidelberg, Germany; ^4^ Translational Lung Research Center Heidelberg (TLRC-H), Member of the German Center for Lung Research (DZL), Heidelberg, Germany; ^5^ Institute of Pathology, University Hospital Heidelberg, Heidelberg, Germany; ^6^ Department of Internal Medicine II, Medical Oncology, University Hospital Würzburg, Würzburg, Germany; ^7^ Klinikum Würzburg Mitte, Missioklinik, Medizinische Klinik—Schwerpunkt Pneumologie und Beatmungsmedizin, Würzburg, Germany; ^8^ Department of Internal Medicine I, Pneumology, University Hospital Würzburg, Würzburg, Germany; ^9^ Comprehensive Cancer Center Augsburg, Faculty of Medicine, University of Augsburg, Augsburg, Germany

## Abstract

**PURPOSE:**

Epidermal growth factor receptor (EGFR) mutations are a main actionable driver in non–small cell lung cancer (NSCLC). However, the clinical significance of catenin beta-1 (CTNNB1) comutations remains unclear. This study evaluated outcomes of patients with EGFR/CTNNB1 comutated NSCLC in a dual-center cohort.

**METHODS:**

A retrospective analysis of 1,804 patients with NSCLC undergoing next-generation sequencing (NGS) in 2019-2024 at University Hospital Würzburg (single-center cohort, including 15 patients with EGFR/CTNNB1 comutations) was complemented with patients with EGFR/CTNNB1 comutated NSCLC receiving first-line osimertinib at the Thoraxklinik Heidelberg (n = 11) to extend and validate initial findings. We assessed clinical outcomes after first-line osimertinib therapy in 90 EGFR-mutated patients with CTNNB1 wild-type (wt) status and 23 with CTNNB1 comutation.

**RESULTS:**

CTNNB1 mutations were identified in 2.0% (36/1,804) of all patients with NSCLC from the single-center cohort, with 41.7% of these also harboring EGFR mutations. Among EGFR-mutant tumors, 7.7% (15/195) exhibited concurrent CTNNB1 mutations. In the dual-center cohort, the objective response rate with first-line osimertinib was 74.4% in CTNNB1-wt (n = 90) and 65.0% in CTNNB1-mutant patients (n = 23; *P* = .38). Notably, CTNNB1 mutations were associated with significantly longer progression-free survival (PFS; hazard ratio [HR], 0.32; *P* < .001) and overall survival (OS; HR, 0.33; *P* = .003). Multivariate analysis confirmed CTNNB1 comutation as an independent prognostic factor for improved PFS (HR, 0.31 [95% CI, 0.14 to 0.69]; *P* = .004) and OS (HR, 0.26 [95% CI, 0.10 to 0.65]; *P* = .004). Additionally, CTNNB1 mutations correlated with lower PD-L1 expression (*P* = .001) and TP53-wt status (*P* < .001).

**CONCLUSION:**

CTNNB1 comutations are associated with lower PD-L1 expression and TP53-wt status, correlating with improved outcomes in patients with EGFR-mutant NSCLC undergoing osimertinib therapy. These results suggest that CTNNB1 comutations may serve as a favorable prognostic biomarker in patients with EGFR-mutant NSCLC. Additional prospective studies are warranted to validate these results.

## INTRODUCTION

Non–small cell lung cancer (NSCLC) accounts for 80%-85% of lung cancer cases and remains a leading cause of cancer-related mortality. Targeted therapies have notably improved outcomes, especially in patients harboring epidermal growth factor receptor (EGFR) mutations.^1^ The prevalence of EGFR mutations is influenced by factors such as adenocarcinoma histology, nonsmoking status, female sex, and ethnicity, with rates ranging from 10%-15% in Western populations to up to 40% in Asian populations.^2^ Common activating EGFR mutations, including in-frame deletions within exon 19 (Ex19del) and the L858R substitution in exon 21, account for 90% of EGFR mutations in NSCLC and are associated with high sensitivity to EGFR tyrosine kinase inhibitors (EGFR-TKIs).^1,3^ Conversely, atypical EGFR mutations may contribute to reduced responsiveness to EGFR-TKIs, underscoring the heterogeneity of therapeutic response among EGFR-mutant NSCLC.^3^ Osimertinib, a third-generation EGFR-TKI, is a standard first-line treatment for patients with common EGFR mutations on the basis of the results from the FLAURA trial.^4^ However, its efficacy is limited by primary and acquired resistance, which highlights the importance of the underlying biological context. Certain molecular features, like TP53 comutations,^5,6^ have been associated with worse outcomes under EGFR inhibitors.

**CONTEXT**

**Key Objective**
Do catenin beta-1 (CTNNB1) comutations influence clinical outcomes in patients with epidermal growth factor receptor (EGFR)–mutant non–small cell lung cancer (NSCLC) receiving EGFR-targeted therapy?
**Knowledge Generated**
In our dual-center analysis, NSCLC patients with EGFR/CTNNB1 comutations receiving first-line osimertinib had significantly improved progression-free and overall survival compared with those patients with CTNNB1 wild-type tumors. CTNNB1 comutations emerged as an independent prognostic factor in EGFR-mutant NSCLC, although these tumors were associated with low PD-L1 expression and TP53 wild-type status.
**Relevance**
Identifying CTNNB1 co-mutations as a marker of improved outcome may help to stratify patients with EGFR-mutated NSCLC and guide therapeutic decision-making in clinical practice.

The *Catenin beta-1* (*CTNNB1*) gene, encoding β-catenin, is involved in the Wingless-related integration site (Wnt) signaling pathway and plays a significant role in various cancers.^7^ An extensive analysis of 7,437 patients with lung adenocarcinoma using the cBioPortal database revealed a significant positive correlation between mutations in EGFR and CTNNB1 (Log2 odds ratio, 1.065; *P* < .001; q < 0.001),^8^ which is consistent with previous studies showing that CTNNB1 mutations frequently co-occur with EGFR mutations in lung adenocarcinoma.^9,10^ Furthermore, in vitro data suggest that CTNNB1 mutations may represent a resistance mechanism to EGFR-targeted therapies.^11-15^ However, the clinical significance of co-occurring CTNNB1 and EGFR mutations in NSCLC remains unclear because current research provides limited information on their impact on treatment efficacy and disease outcomes.^9,16-18^

## METHODS

### Data Characteristics

For the single-center cohort, next-generation sequencing (NGS) data of 1,804 patients with unresectable and advanced NSCLC who received molecular diagnostics by the National Network Genomic Medicine (nNGM) Lung Cancer center Würzburg^19^ from January 2019 to March 2024 were retrospectively analyzed. For patients receiving more than one molecular testing during this time period, only the first molecular testing was included in the analysis. Histopathologic diagnosis, PD-L1 assessment, and panel-based molecular testing were performed. The nNGM lung cancer panel for DNA-based NGS included, depending on the panel version, between 19 and 28 genes to detect mutations, including *CTNNB1*, *EGFR*, *KRAS*, *BRAF*, *ERBB2*, and *MET* (Appendix Table A1). Gene rearrangements/fusions in *ALK*, *RET*, and *ROS1* as well as amplifications in *MET* were detected by FISH and/or RNA-based NGS. To evaluate the impact of CTNNB1 comutations on EGFR-TKI therapy outcomes, a comparative analysis was performed. To extend and validate initial findings, data of all EGFR-mutated patients with concurrent CTNNB1 mutation receiving first-line osimertinib during the same time period 2019-2023 in the Thoraxklinik at Heidelberg University Hospital (n = 11) were added to form a dual-center cohort of EGFR-mutated stage IV NSCLC. These patients had been identified using combined DNA/RNA NGS as published.^20^ In the dual-center cohort, patients were permitted to have a documented history of curative intended treatment for their NSCLC, initial chemotherapy as bridging therapy, or radiotherapy for metastases during palliative first-line treatment. The study adhered to the ethical standards set by the Declaration of Helsinki and received approval from the ethics committee of the Medical Faculty at the University of Würzburg (no. 221/19_z) and Heidelberg (S-469/2017), which waived the need for informed consent because of its retrospective character.

### Statistical Analysis

Objective response rate (ORR) and disease control rate (DCR) were calculated by the patients' best objective response during the treatment period. The ORR included patients who achieved either complete response (CR) or partial response (PR), whereas the DCR included patients who achieved CR, PR, or stable disease (SD). The median follow-up time was calculated according to the reverse Kaplan-Meier method.^20^ For progression-free survival (PFS), the progression date was verified with review of radiologic images, that is, chest computed tomography/brain MRI every 6-12 weeks, by the investigators without formal RECIST re-evaluation. PFS was calculated from the day of initiation of the targeted therapy until disease progression or death. Overall survival (OS) was calculated from the day of the initial NSCLC diagnosis in the palliative setting to death. Kaplan-Meier analysis was used to compare the PFS and OS. Patients without progression or patients still alive at the time of last follow-up were censored. A log-rank test was applied to analyze the differences between the Kaplan-Meier curves. Multivariable Cox regression was performed to assess the relationship between multiple independent variables and the PFS and OS. Hazard ratios (HRs) with 95% CIs for each covariate included in the model were provided. Statistical significance was determined at a *P* < .05. The Chi-square test was used to assess the independence of variables. The Phi coefficient was applied to determine the strength of the correlation. The Fisher exact test was used when the expected frequency in any category was <5. Statistical analyses were performed using IBM SPSS statistics version 29.0 (IBM, Armonk, NY). Graphs were created with GraphPad Prism version 10.1.2 (GraphPad Software, Boston, MA).

## RESULTS

**FIG 1.**
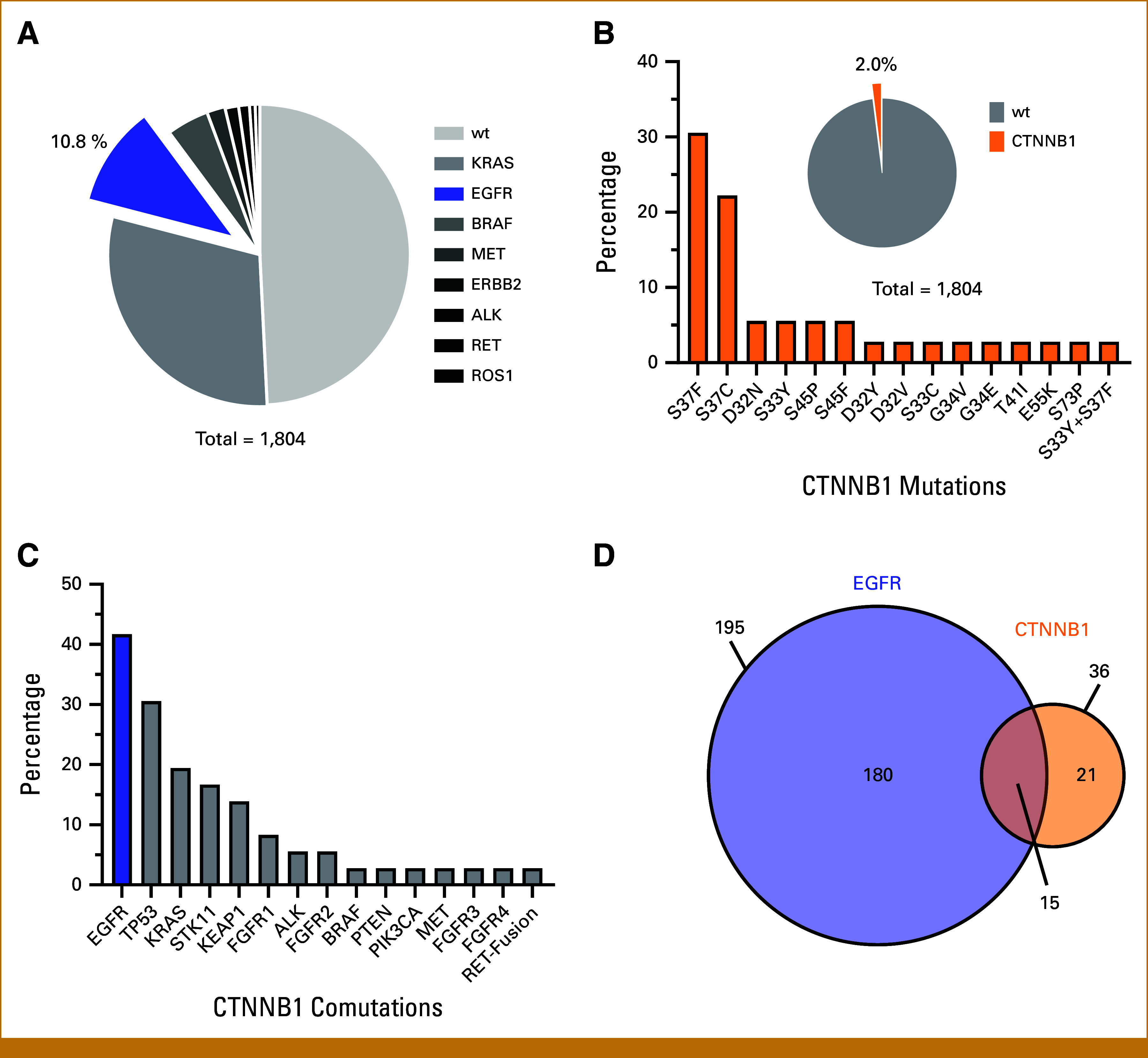
Co-occurrence of EGFR and CTNNB1 mutations in patients with NSCLC. (A) Distribution of key oncogenic driver mutations within a single-center cohort of 1,804 patients with NSCLC. (B) Detailed analysis of activating CTNNB1 mutations, showing their frequency and types across the study population (n = 36). (C) Comutation patterns involving CTNNB1 in NSCLC, with a focus on the notable prevalence of concurrent EGFR mutations. (D) Venn diagram showing the overlap between EGFR and CTNNB1 mutations (n = 15) in the single-center cohort. *P* < .0001 for the frequency of CTNNB1 in EGFR-mutant versus unselected NSCLC with a chi-square test. CTNNB1, catenin beta-1; EGFR, epidermal growth factor receptor; NSCLC, non–small cell lung cancer.

This study offers a comprehensive analysis of the incidence and treatment outcomes in patients with co-occurring EGFR and CTNNB1 mutations. Initially, we assessed the incidence and molecular characteristics of CTNNB1 mutations in NSCLC. A retrospective single-center analysis was conducted at the University Hospital Würzburg, encompassing 1,804 patients with NSCLC from January 2019 to March 2024. Genomic data were analyzed using a NGS panel to identify co-occurring genomic aberrations, focusing on actionable genes. Within this cohort, 195 patients (10.8%) harbored EGFR mutations, consisting of 96 EGFR Ex19del, 38 L858R, 34 atypical, and 27 complex mutations. The distribution of key oncogenic driver mutations across the cohort is shown in Figure 1A. CTNNB1 mutations were identified in 2.0% of patients (n = 36), primarily located in exon 3. These hot spot mutations impair key phosphorylation sites leading to the stabilization and nuclear accumulation of β-catenin (Fig 1B). Compared with other key drivers, CTNNB1 mutations were significantly associated with EGFR mutations, which occurred in 41.7% (n = 15) of patients with CTNNB1-mutated NSCLC (Fig 1C). In line with results from previous studies,^17^ the prevalence of CTNNB1 mutations was higher in EGFR-mutated (7.7%) than in EGFR wild-type NSCLC (1.3%; χ^2^(1) = 36.279; *P* < .0001; Fig 1D).

Table 1 summarizes the clinical and genetic characteristics, treatment regimens, and outcomes of 15 patients with NSCLC with co-occurring EGFR and CTNNB1 mutations in the single-center cohort. All patients had adenocarcinoma, and CTNNB1 mutations were detected at initial diagnosis, predominantly as subclonal alterations (73.3%, n = 11/15). The majority harbored an EGFR Ex19del (66.67%, n = 10) or a L858R point mutation (20.0%, n = 3), with two patients having a complex EGFR mutation consisting of a L858R together with an uncommon mutation (patient 1) or two uncommon mutations (patient 5). Additional coalterations included mutations in TP53, FGFR1, FGFR2, PIK3CA, or PTEN as well as high-level MET amplification.

**TABLE 1.** Patient Characteristics and Treatment Outcomes in EGFR-Mutant NSCLC With Co-Occurring CTNNB1 Mutations

ID	Age	Sex	Histology	UICC	EGFR Mutation (frequency)	CTNNB1 Mutation (frequency)	Other Genetic Alterations	Pretreatment	TKI	Line of Therapy (palliative)	Best Response	PFS (mos)	OS (mos)
Pat 1	78	M	ADC	IVb	L858R (5%) + T854A (5%)	S45F (8%)	—	No	Osimertinib	1st line	PR	39.2	50
Pat 2	63	M	ADC	IVb	Ex19del (40%)	S37C (18%)	FGFR2 L526R, PIK3CA G1049R, TP53 c.673-1G>A p.?	No	Osimertinib	1st line	PR	15.3	50.9
Pat 3	72	M	ADC	IVa	Ex19del (8%)	S37F (2%)	—	No	Osimertinib	1st line	PR	54.8+	55.1+
Pat 4	78	F	ADC	IIIc	Ex19del (98%)	D32N (24%)	FGFR1 L172R, TP53 L289fs, high-level MET amplification (polysomy)	Yes, RCT (CIS/VIN)	Afatinib	1st line	PR	41.0	47.1
Pat 5	72	M	ADC	IVb	G719A (27%) + S768I (28%)	S45P (0.4%)	PTEN N117Ifs*17	No	Osimertinib	1st line	SD	7.1	10.9
Pat 6	75	F	ADC	IVb	Ex19del (53%)	S37C (14%)	—	No	Osimertinib	1st line	PR	15.0	34,6+
Pat 7	82	F	ADC	IVb	L858R (94%)	S33C (5%)	—	No	Osimertinib	1st line	PD	3.8	12.0
Pat 8	78	M	ADC	IVa	Ex19del (33%)	G34V (13%)	—	Yes, SUR	Osimertinib	1st line	nd	37.0+	39.0+
Pat 9	70	F	ADC	IVb	Ex19del (22%)	S37F (13%)	—	No	Osimertinib	1st line	PR	26.5+	27.5+
Pat 10	68	F	ADC	IVb	Ex19del (44%)	S37F (25%)	—	Yes, CAR/PAC	Osimertinib	2nd line	PR	21.3+	30.4+
Pat 11	80	F	ADC	IVa	Ex19del (29%)	S37C (8%)	—	No	Osimertinib	1st line	SD	24.4+	25.0+
Pat 12	69	M	ADC	IVb	Ex19del (19%)	S33C (19%)	—	No	Osimertinib	1st line	PR	17.1+	18.1+
Pat 13	84	F	ADC	IVa	L858R (39%)	D32N (7%)	—	Yes, SUR	Not yet	—	—	—	—
Pat 14	52	F	ADC	IVa	Ex19del (52%)	S33Y (57%)	FGFR1 R601Q	No	Osimertinib	1st line	PR	9.1+	9.1+
Pat 15	73	F	ADC	IVa	L858R (29%)	S33Y (25%)	TP53 R249_P250delinsSS, high-level MET amplification (polysomy)	No	Osimertinib	1st line	SD	5.9+	6.9+

NOTE. Detailed overview of the patient characteristics and clinical outcomes in the single-center cohort of patients with EGFR-mutant NSCLC with concurrent CTNNB1 mutations. Demographic data, including age, sex, and histological subtypes, are presented along with genetic profiles revealing EGFR and CTNNB1 mutations as well as additional genetic alterations in patients. Treatment modalities, such as the type of TKI administered and the line of therapy, are described. The figure also includes clinical outcomes, highlighting the BOR to therapy, PFS, and OS in months, illustrating the different responses to treatment in patients with complex mutational landscapes.

Abbreviations: ADC, adenocarcinoma; BOR, best objective response; CAR/PAC, carboplatin/paclitaxel; CIS/VIN, cisplatin/vinorelbine; CTNNB1, Catenin beta-1; EGFR, epidermal growth factor receptor; Ex19del, in-frame deletion within exon 19; F, female; M, male; mos, months; nd, not defined; OS, overall survival; Pat, patient; PD, progressive disease; PFS, progression-free survival; PR, partial response; RCT, radiochemotherapy; SD, stable disease; SUR, surgery; TKI, tyrosine kinase inhibitor; UICC, Union for International Cancer Control; +, indicates that disease progression under therapy or death of the patient did not occur at the time point of last follow-up.

For therapy, osimertinib was the primary first-line palliative therapy (80.0%, n = 12/15), with one patient receiving the second-generation EGFR-TKI afatinib and another receiving osimertinib as second-line palliative therapy. Three patients underwent curative therapy (surgery: n = 2, radiochemotherapy: n = 1). The ORR to EGFR-TKIs was 69.2% (n = 9/13), and the DCR was 92.3% (n = 12/13). Response to osimertinib in patient 8 was indeterminate because of the lack of measurable tumor lesions postsurgery, and patient 13 had not yet started palliative therapy. At the last follow-up examination, disease progression occurred in six patients undergoing targeted therapy.

Notably, in patient 5, a tumor rebiopsy revealed a significant increase in CTNNB1 mutation frequency (0.4%-30.0%), alongside an increase in EGFR allele frequency (27/28% to 62/62%), correlating with a higher tumor cell content (40% *v* 70% at rebiopsy). Despite this, the patient opted to continue osimertinib until death. By contrast, the CTNNB1 mutation could not be detected in rebiopsy of patient 4 after progressing on afatinib. This is likely to be a result of the extremely limited quantity of tumor sample examined, which corresponds to the low allele frequency of 8% of the EGFR mutation. At the time of the initial diagnosis, the allele frequency of the EGFR mutation was 98%, and the CTNNB1 mutation had an allele frequency of 24%. Patients 1 and 7 declined rebiopsy, and patient 2 was excluded because of deteriorating health. Patient 6, with cerebral progression, received radiation therapy.

To assess the impact of CTNNB1 comutations on EGFR-TKI therapy outcomes, a comparative analysis was performed. To increase statistical power and ensure a sufficiently large case cohort, additional data from patients with EGFR-mutant NSCLC harboring CTNNB1 comutations were collected from the Thoraxklinik at University Hospital Heidelberg (2019-2023) and integrated into a dual-center data set. The comparative analysis of the dual-center cohort included 90 EGFR-mutant patients with wild-type CTNNB1 (CTNNB1-wt) and 23 with CTNNB1 comutations (CTNNB1-mut). Detailed characteristics provided in Appendix Table A2.

**FIG 2.**
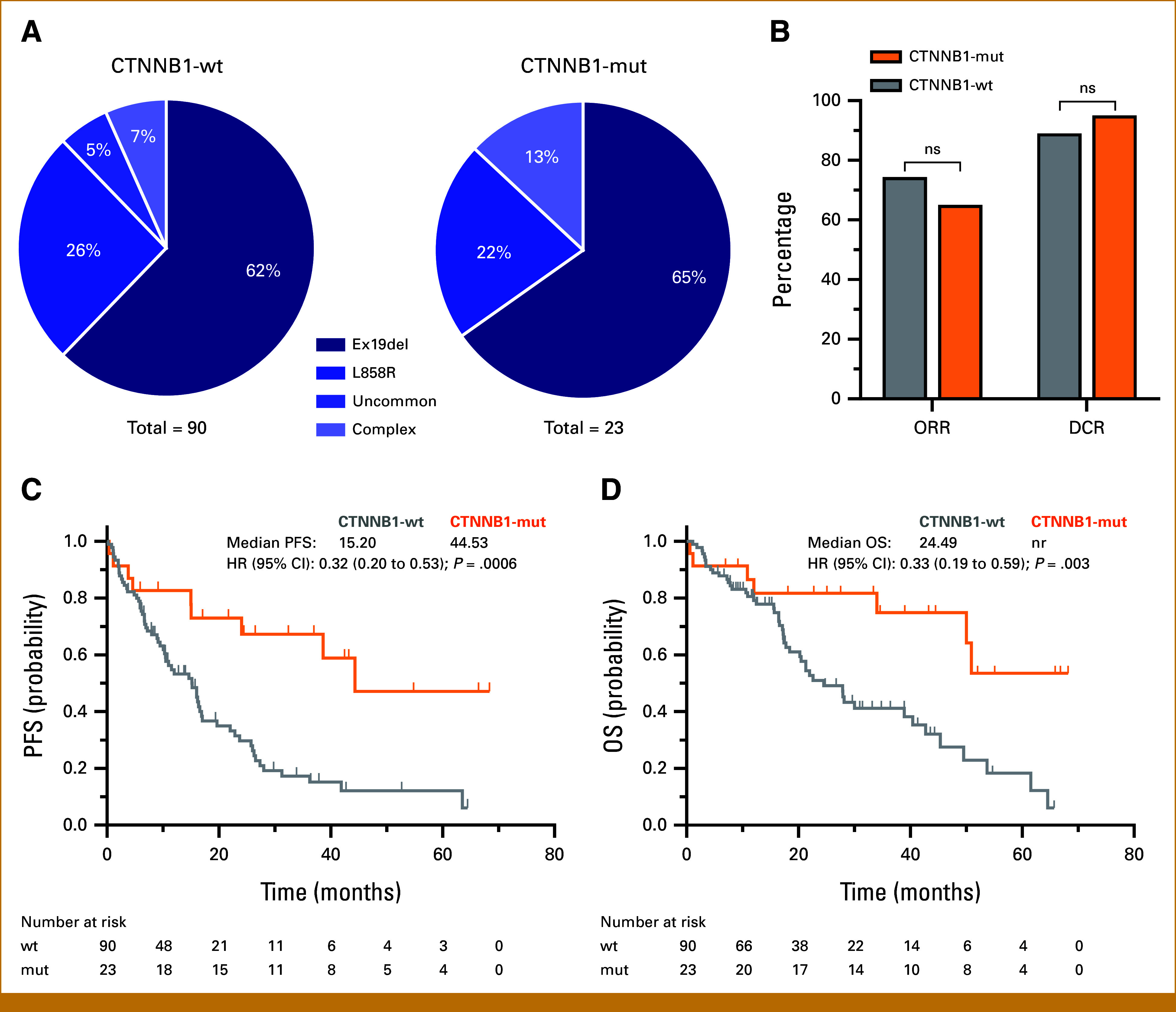
CTNNB1 comutations are associated with improved clinical outcomes in patients with EGFR-mutant NSCLC. (A) Distribution of EGFR Ex19del, EGFR L858R mutations, and uncommon or complex EGFR mutations in CTNNB1 wild-type (CTNNB1-wt) and CTNNB1 mutant (CTNNB1-mut) patients. (B) Comparative analysis of the ORR and DCR between CTNNB1-wt and CTNNB1-mut groups. Chi-square test confirmed no significance of ORR (*P* = .399) and DCR (*P* = .420) between CTNNB1-wt and CTNNB1-mut patient group. (C, D) Kaplan-Meier survival curves comparing PFS and OS between CTNNB1-wt and CTNNB1-mut groups. Log-rank test indicate statistically significant differences in both PFS and OS between the two groups. CTNNB1, catenin beta-1; DCR, disease control rate; EGFR, epidermal growth factor receptor; Ex19del,  in-frame deletion within exon 19; HR, hazard ratio; nr, not reached; ns, no significance; ORR, objective response rate; OS, overall survival; PFS, progression-free survival.

Among the CTNNB1-wt group, 62.2% of patients had an EGFR Ex19del, 25.6% had a L858R mutation, and 12.3% harbored uncommon or complex EGFR mutations. The CTNNB1-mut group demonstrated a comparable pattern, with 65.2%, 21.7%, and 13.0%, respectively (Fig 2A). Osimertinib yielded an ORR of 74.4% in CTNNB1-wt patients versus 65.0% in CTNNB1-mut patients (χ^2^(1) = 0.712; *P* = .399) and a DCR of 89.0% and 95.0% (χ^2^(1) = 0.649; *P* = .420), respectively (Fig 2B). In the CTNNB1-wt group, 16.7%, 61.1%, and 22.2% had PD-L1 expression <1%, 1%-49%, and ≥50%, respectively. By contrast, the CTNNB1-mut group showed different proportions: 52.2%, 43.4%, and 4.3%. The Fisher exact test showed significant relationship between CTNNB1 mutation status and PD-L1 tumor proportion score (TPS) when categorized as <1%, 1%-49%, and ≥50% (χ^2^(2) = 13.805; *P* = .001), confirming the observation that CTNNB1-mutated patients by mean had a lower PD-L1 TPS. Moreover, the CTNNB1-wt group had a higher frequency of TP53 comutations compared with the CTNNB1-mut group (58.9% *v* 13.0%). A chi-square test was used to compare CTNNB1 and TP53 mutational status. The results showed a negative correlation between CTNNB1 and TP53 mutations in the cohort (χ^2^(1) = 15.402; *P* < .001; φ = –0.369). To confirm this relationship, an analysis of 12,261 patients with NSCLC from the cBioPortal database showed mutual exclusivity of CTNNB1 and TP53 (Log2 odds ratio, –0.663; *P* < .001; q < 0.001),^8^ suggesting that this association is not limited to EGFR-mutated patients. Moreover, a reduced proportion of patients in the CTNNB1-mut group exhibited MET amplification (17.8% *v* 4.3%). However, no significant correlation between CTNNB1 mutation status and MET amplification was indicated by the Fisher exact test (χ^2^(1) = 1.824; *P* = .0.177).

**FIG A1.**
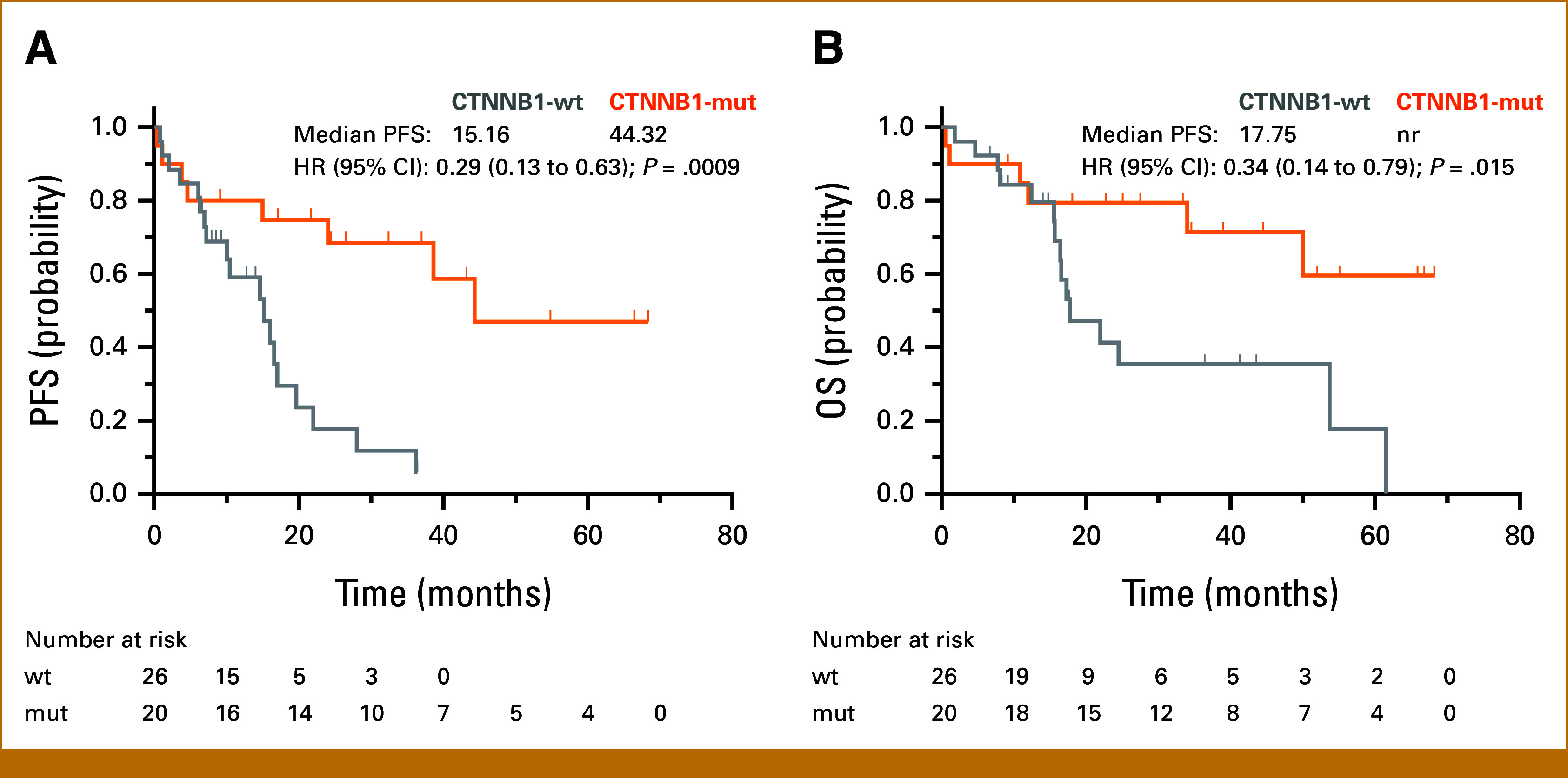
Clinical outcomes of EGFR-mutant NSCLC patients without TP53 comutation and PD-L1 TPS <50%. Subgroup analysis of EGFR-mutant NSCLC patients without TP53 comutation and PD-L1 TPS <50%, stratified by CTNNB1 mutation status (CTNNB1-wt: n = 26; CTNNB1-mut: n = 20). (A) Kaplan-Meier curve for PFS. (B) Kaplan-Meier curve for OS. Statistical significance was assessed using the log-rank test, with *P* < .05 considered significant. CTNNB1, catenin beta-1; EGFR, epidermal growth factor receptor; HR, hazard ratio; nr, not reached; NSCLC, non–small cell lung cancer; OS, overall survival; PFS, progression-free survival; TPS, tumor proportion score.

The median follow-up time was 31.46 months for the CTNNB1-wt and 43.27 months for the CTNNB1-mut group. Kaplan-Meier analyses demonstrated significant differences in PFS and OS between the groups (Figs 2C and 2D). The median PFS was 15.2 months for the CTNNB1-wt group and 44.53 months for the CTNNB1-mut group (HR, 0.32 [95% CI, 0.20 to 0.53]; *P* = .0006). Similarly, the median OS was 24.49 months for the CTNNB1-wt group, whereas the CTNNB1-mut group had not reached median OS (HR, 0.33 [95% CI, 0.19 to 0.59]; *P* = .003). Given that the CTNNB1-mutant group exhibited a lower prevalence of TP53 mutations and lower PD-L1 TPS compared with the CTNNB1-wt group, we conducted a subgroup analysis focusing on TP53-wt patients with PD-L1 TPS <50%. Stratification by CTNNB1 mutation status revealed a significant improvement in PFS (HR, 0.29 [95% CI, 0.13 to 0.63]; *P* = .0009) and OS (HR, 0.34 [95% CI, 0.14 to 0.79]; *P* = .015) in CTNNB1-mut patients (n = 20) compared with CTNNB1-wt patients (n = 26; Appendix Fig A1).

**FIG 3.**
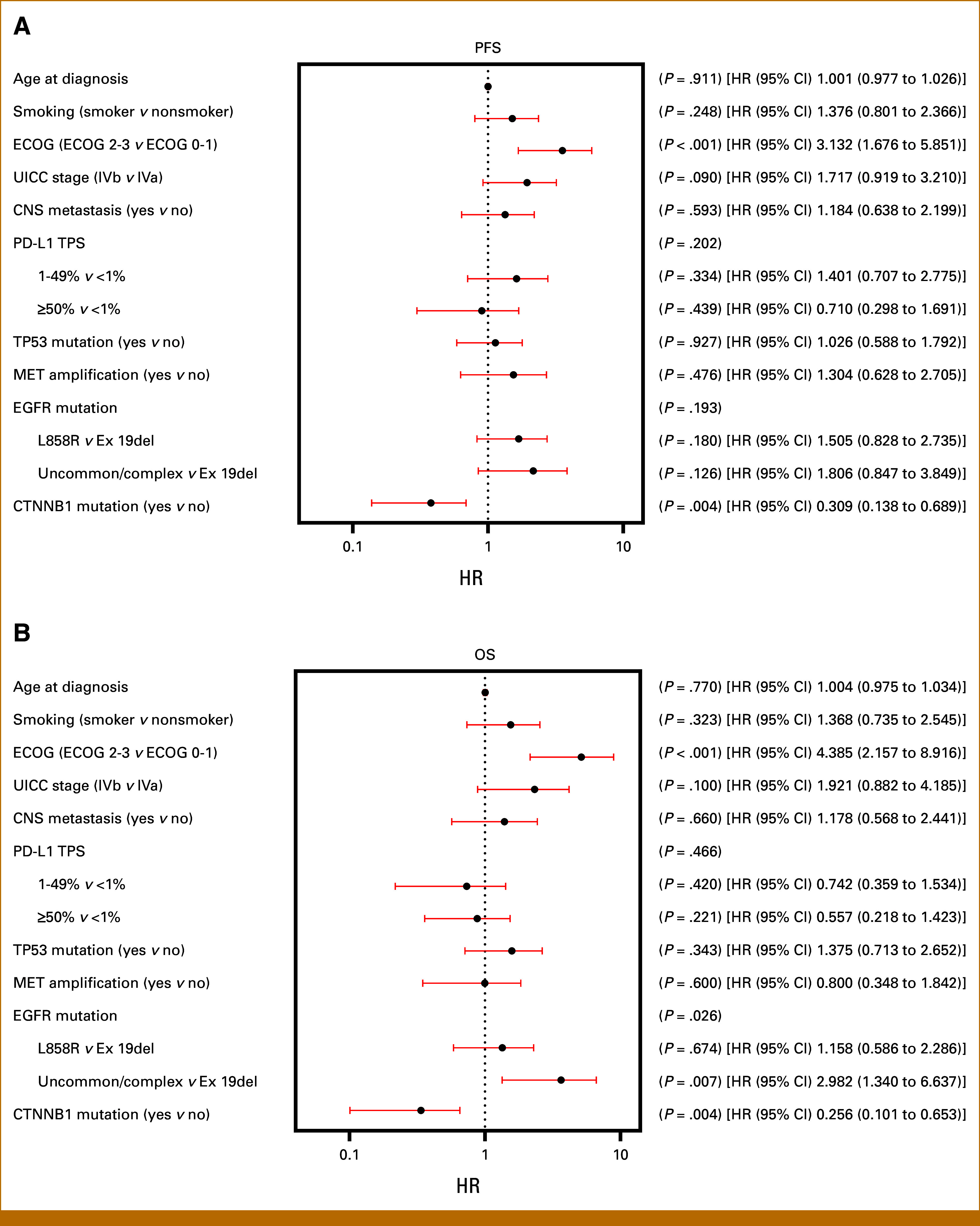
Forest plots depicting the cox proportional hazards regression analysis for clinical outcomes in patients with EGFR-mutant NSCLC. (A) Analysis of factors influencing PFS. HRs are represented by black dots, with red whiskers indicating the 95% CI. Key factors analyzed include ECOG performance status, UICC stage at the time of palliative first-line therapy, CNS metastasis, PD-L1 TPS, TP53 mutation status, MET amplification, and the presence of specific EGFR and CTNNB1 mutations. Notably, poor ECOG performance status is significantly associated with shorter PFS, whereas the presence of co-occurring CTNNB1 mutations is associated with improved PFS. (B) Analysis of factors that affect OS. Similar to the PFS analysis, HRs are represented with 95% CIs. Significant predictors of reduced OS include poor ECOG performance status and uncommon or complex EGFR mutations. Conversely, concurring CTNNB1 mutation is associated with improved OS. CTNNB1, catenin beta-1; ECOG, Eastern Cooperative Oncology Group; EGFR, epidermal growth factor receptor; HR, hazard ratio; NSCLC, non–small cell lung cancer; OS, overall survival; PFS, progression-free survival; TPS, tumor proportion score; UICC, Union for International Cancer Control.

**FIG A2.**
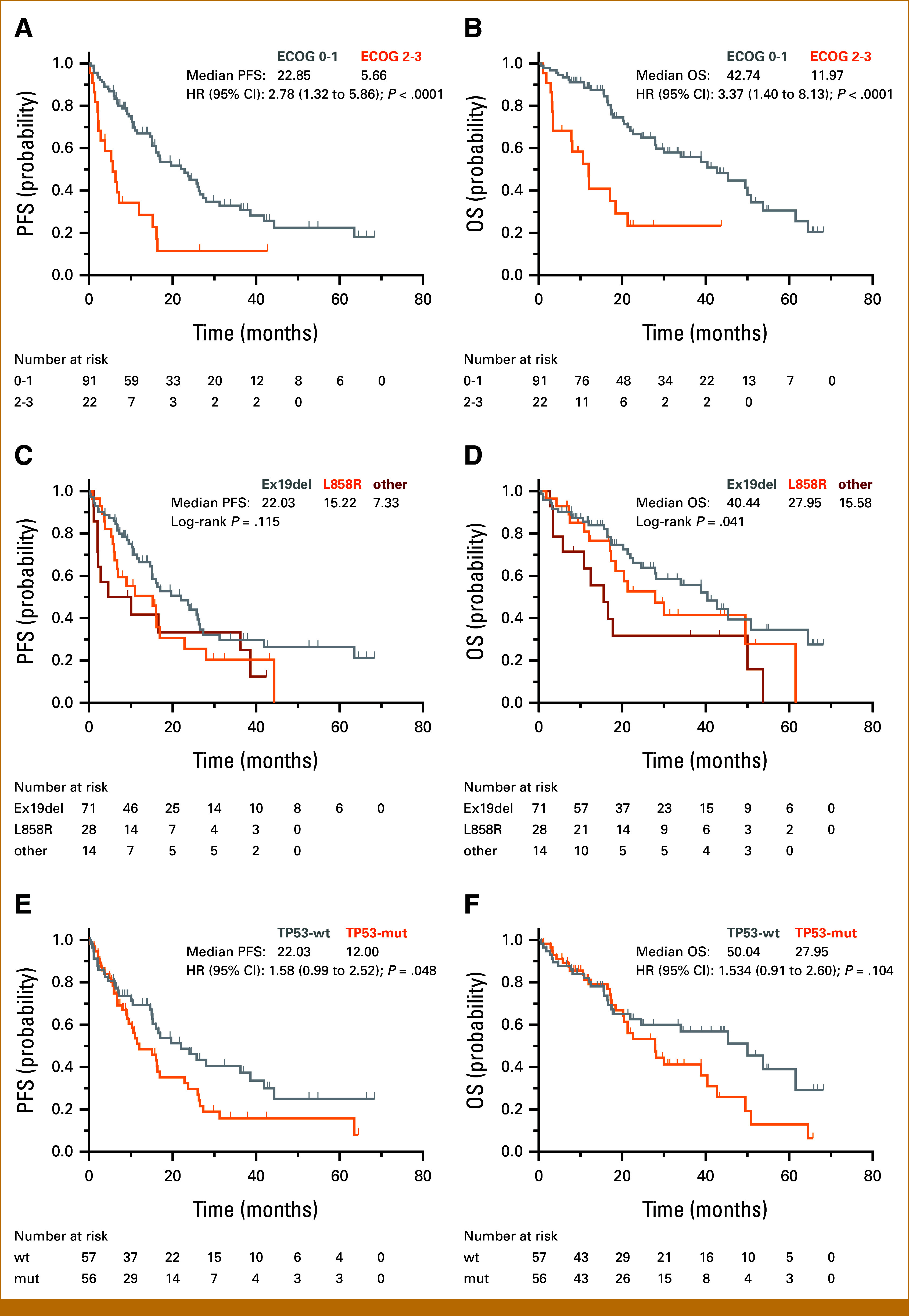
Clinical outcomes of patients with EGFR-mutant NSCLC on the basis of ECOG performance status, EGFR mutation subtypes, and TP53 mutation status. (A, C and E) illustrate Kaplan-Meier curves for PFS on the basis of ECOG performance status, EGFR mutation subtypes (Ex19del, L858R and uncommon/complex EGFR mutations) and TP53 mutation status. (B, D and F) display Kaplan-Meier curves for OS on the basis of ECOG performance status, EGFR mutation subtypes and TP53 mutation status. A log-rank test was conducted to assess statistical significance, with a *P* value <.05 considered significant. ECOG, Eastern Cooperative Oncology Group; EGFR, epidermal growth factor receptor; Ex19del, in-frame deletion within exon 19; HR, hazard ratio; NSCLC, non–small cell lung cancer; OS, overall survival; PFS, progression-free survival.

To determine whether CTNNB1 is an independent prognostic marker, we conducted a multivariable Cox regression analysis. Therefore, we assessed the impact of several variables, including age, smoking history, ECOG performance status, Union for International Cancer Control stage, CNS metastasis, PD-L1 expression, TP53 mutation status, MET amplification, EGFR mutation subtypes, and CTNNB1 mutation status on both PFS and OS (Fig 3). Consistent with the univariable analysis, the presence of CTNNB1 comutations emerged as a significant prognostic factor for improved PFS (HR, 0.31 [95% CI, 0.14 to 0.69]; *P* = .004) and OS (HR, 0.26 [95% CI, 0.10 to 0.65]; *P* = .004). Poor ECOG performance status was identified as predictor of poor PFS (HR, 3.13 [95% CI, 1.68 to 5.85]; *P* < .001) and OS (HR, 4.39 [95% CI, 2.16 to 8.92]; *P* < .001). Additionally, uncommon and complex EGFR mutations were significantly associated with reduced OS (HR, 2.98 [95% CI, 1.34 to 6.64]; *P* = .007). In the multivariable analysis, TP53 was not identified as a significant prognostic factor for either PFS or OS when analyzed alongside CTNNB1. Conversely, in the univariable analysis, patients with TP53 comutation exhibited significantly poorer PFS (HR, 1.58 [95% CI, 0.99 to 2.52]; *P* = .048), whereas a trend was observed for OS (HR, 1.53 [95% CI, 0.91 to 2.60]; *P* = .104), although it did not reach statistical significance. Kaplan-Meier curves for ECOG performance status, EGFR mutation types, and TP53 mutation status are shown in Appendix Fig A2.

## DISCUSSION

This study is one of the most comprehensive retrospective analyses of patients with EGFR-mutant NSCLC with co-occurring CTNNB1 mutations. The most important finding of this study was the improved clinical outcome associated with CTNNB1 comutations when present at baseline in EGFR-driven lung cancer. Despite a similar ORR compared with CTNNB1-wt patients, CTNNB1-mut patients achieved superior and prolonged disease control, as well as significantly improved PFS and OS with osimertinib treatment. Although the expression of β-catenin has been described to be increased in EGFR-mutant NSCLC,^14^ an additional potential increase in Wnt pathway activity because of pathogenic CTNNB1 mutations had no impact on patient outcome. Instead, our data indicate that CTNNB1 comutations may have a protective effect, leading to improved PFS and OS, challenging the traditional view that additional oncogenic mutations uniformly worsen outcomes. This raises questions about the potential benefit of combining EGFR TKIs with β-catenin inhibitors to enhance clinical outcomes in EGFR-mutant NSCLC.^21^ This is in contrast to previous studies that focused on the role of CTNNB1 as possible resistance mechanism or lacked sufficient clinical data.^9,11-18^

Of note, this study is one of the largest studies analyzing CTNNB1 mutations in EGFR-mutated NSCLC and, to our knowledge, the first one that systematically investigates the response to EGFR-TKIs in first-line therapy. Although CTNNB1 mutations are rare in EGFR-mutated NSCLC, our dual-center approach enabled a robust and meaningful analysis. The cohort analyzed here consisted mainly of elderly patients with adenocarcinoma, a typical population group for EGFR-mutant NSCLC. Our findings confirm the prognostic value of ECOG performance status, where higher scores correlate with significantly shorter PFS and OS. In addition, the presence of uncommon and complex EGFR mutations was associated with poorer OS, another typical finding underscoring the representative nature of our cohort and the therapeutic challenges of standard EGFR-TKI therapy for NSCLC with these mutations. Notably, CTNNB1 mutations frequently co-occurred together with both classical and atypical EGFR alterations.

Although Wnt/β-catenin signaling is typically implicated in tumorigenesis,^7^ its role in this context may involve interactions with effective EGFR-TKI treatment leading to transcriptional downregulation of CTNNB1 expression.^22^ Moreover, certain cell types, such as bronchiolar epithelial cells, may be inherently resistant to its oncogenic effects.^23^ However, in the context of frank disease progression, it is conceivable that the β-catenin levels may contribute to the resistance network in drug-sensitive/resistant cells. These observations may explain the improved clinical outcome in patients with CTNNB1 comutations and support the continued use of EGFR-TKIs regardless of CTNNB1 mutation status. Notably, a similar protective effect of CTNNB1 mutations has also been observed in endometrial carcinoma, indicating this may not be specific to NSCLC.^24^

Our analysis revealed a negative correlation between CTNNB1 and TP53 mutations in the comutational landscape of EGFR-mutant NSCLC, supported by data from the cBioPortal database. As TP53 mutations have been linked to poorer outcomes with EGFR-targeted therapies,^5,6^ including third-generation TKI osimertinib,^25-27^ the anti-correlation with CTNNB1 mutations represents a potential explanation of the apparently favorable impact of the latter in this analysis. In addition, it may explain the lack of statistical significance for PFS and OS with TP53, when analyzed alongside CTNNB1 status in multivariable regression. Furthermore, our findings suggest that although TP53 mutations are often considered a negative prognostic factor in EGFR-mutant NSCLC, their impact may be context-dependent and influenced by additional comutations, such as CTNNB1.

Additionally, we identified a significant correlation between CTNNB1 mutation status and lower PD-L1 TPS. This finding is noteworthy because high PD-L1 expression has been previously linked to shorter PFS and OS under first-line osimertinib treatment.^28-31^ Of note, a higher PD-L1 expression has also been linked to more aggressive courses of driver-dependent NSCLC, for example, ALK+ and METΔex14+ tumors,^32,33^ and may reflect a stronger oncogenic signaling, as PD-L1 is a downstream target of these cascades. The link between CTNNB1 mutations and reduced PD-L1 expression also suggests a potential interaction between the Wnt/β-catenin signaling pathway and immune evasion in EGFR-mutant NSCLC. Previous studies have implicated Wnt/β-catenin activation in modulating immune cell infiltration within the tumor microenvironment.^34^ Our findings align with this concept, potentially indicating that CTNNB1-mutant tumors may exhibit distinct immunobiologic properties. Additionally, CTNNB1-mutant patients showed a lower prevalence of MET amplification, although the association was not statistically significant. Although MET amplification is a well-established resistance mechanism to osimertinib,^35^ its role as an intrinsic driver in EGFR-TKI therapy remains inadequately understood. Despite these findings, neither PD-L1 TPS nor MET amplification proved to be significant predictors in the multivariable analysis.

Collectively, patients with CTNNB1 mutations exhibit molecular features indicative of a favorable prognosis, including the absence of TP53 mutations and low PD-L1 TPS, and a tendency to have less MET amplification. Importantly, a subgroup analysis of TP53-wt patients with a PD-L1 TPS <50% in the dual-center cohort demonstrated significantly better PFS and OS for CTNNB1-mut patients. These findings suggest that CTNNB1 may be an independent prognostic factor and that better clinical outcomes may not solely be caused by the TP53 or PD-L1 status.

In conclusion, our findings demonstrate that concurrent CTNNB1 mutations may serve as independent prognostic markers for improved clinical outcomes in EGFR-mutated NSCLC. It is important to note that the retrospective nature of the study and the relatively small sample size limit the generalizability of these findings. The lack of prospective validation necessitates a cautious interpretation of the results. To substantiate these findings and better understand the mechanistic basis of the survival benefit associated with CTNNB1 mutations, larger, prospectively designed cohorts and multicenter studies are essential to improve data diversity and enhance the generalizability.

## APPENDIX

**TABLE A1.** Genes and Exon Coverage in the nNGM Lung Cancer Panels

Gene	nNGM-Panel 1.0	nNGM-Panel 2.0	nNGM-Panel 3.1
*ALK*	Exons: 22-25		Exons: 20-28
*BRAF*	Exons: 11, 15		Exons: 11, 12, 14, 15
*CTNNB1*	Exons: 3		
*CUL3*			Exons: 1-16
*EGFR*	Exons: 18-21		
*ERBB2*	Exons: 8, 19, 20		Exons: 8, 18-21
*FGFR1*	Exons: 4-7, 10, 12-15	Exons: 5-8, 11, 13-16	Exons: 5-8, 11, 13-17
*FGFR2*	Exons: 6-15, 18		
*FGFR3*	Exons: 3, 6, 7, 9, 10, 12, 14, 16, 18		Exons: 3, 6, 7, 9, 10, 12, 13-16, 18
*FGFR4*	Exons: 3, 6, 9, 12, 13, 15, 16		
*HRAS*		Exons: 2-4	
*IDH1*	Exons: 4		
*IDH2*	Exons: 4		
*KEAP1*		Exons: 2-6	
*KRAS*	Exons: 2-4		
*MAP2K1*	Exons: 2, 3		
*MET*	Exons: 14 (+introns), 16-19		
*NFE2L2*			Exons: 1-5
*NRAS*	Exons: 2-4		
*NTRK1*		Exons: 13-17	
*NTRK2*		Exons: 16-21	Exons: 14-19
*NTRK3*		Exons: 15-20	
*PIK3CA*	Exons: 10, 21	Exons: 8, 10, 21	
*PTEN*	Exons: 1-8		Exons: 1-8, 9 (without R378-D386)
*RET*		Exons: 10-18	
*ROS1*	Exons: 34-41		
*STK11*		Exons: 1-9	
*TP53*	Exons: 4-8		Exons: 2-11

NOTE. Genes and corresponding exons analyzed across three iterations of the nNGM Lung Cancer Panels, used for DNA-based NGS in NSCLC. The individual panel versions (1.0, 2.0, and 3.1) are compared with each other to demonstrate the progress and expansion of genomic coverage over time. The table lists key oncogenes and tumor suppressor genes, showing the specific exons targeted for mutation detection in each panel. This detailed comparison highlights the panels' increasing ability to detect a wide array of actionable genetic alterations, critical for developing personalized therapeutic approaches in NSCLC.

Abbreviations: NGS, next-generation sequencing; nNGM, National Network Genomic Medicine; NSCLC, non–small cell lung cancer.

**TABLE A2.** Demographic and Clinical Characteristics of EGFR-Mutant NSCLC Patients With and Without CTNNB1 Mutations

Characteristic	Total population	CTNNB1-wt	CTNNB1-mut
No. of patients	113	90	23
Age at diagnosis (mean)	68.69	67.92	71.26
	**No. (%)**	**No. (%)**	**No. (%)**
Sex			
Male	40 (35.4)	33 (36.7)	7 (30.4)
Female	73 (64.6)	57 (63.3)	16 (69.6)
Smoking status			
Nonsmoker	63 (55.8)	49 (54.4)	14 (60.9)
Former smoker	36 (31.9)	30 (33.3)	6 (26.1)
Current smoker	13 (11.5)	10 (11.1)	3 (13.0)
Unknown	1 (0.9)	1 (1.1)	—
Initial curative treatment			
No	101 (89.4)	82 (91.1)	19 (82.6)
Yes	12 (10.6)	8 (8.9)	4 (17.4)
ECOG 1L palliative			
0	35 (31.0)	26 (28.9)	9 (39.1)
1	56 (49.6)	44 (48.9)	12 (52.2)
2	15 (13.3)	14 (15.6)	1 (4.3)
3	7 (6.2)	6 (6.7)	1 (4.3)
UICC-stage 1L palliative			
IVa	37 (32.7)	27 (30.0)	10 (43.5)
IVb	76 (67.3)	63 (70.0)	13 (56.5)
Histology			
Nonsquamous	109 (96.5)	86 (95.6)	23 (100.0)
Squamous	2 (1.8)	2 (2.2)	—
Adenosquamous	2 (1.8)	2 (2.2)	—
CNS metastasis			
No	73 (64.6)	58 (64.4)	15 (65.2)
Yes	40 (35.4)	32 (35.6)	8 (34.8)
PD-L1 TPS			
<1%	27 (23.9)	15 (16.7)	12 (52.2)
1%-49%	65 (57.5)	55 (61.1)	10 (43.5)
≥50%	21 (18.6)	20 (22.2)	1 (4.3)
TP53 mutation status			
Wild-type	57 (50.4)	37 (41.1)	20 (87.0)
Mutated	56 (49.6)	53 (58.9)	3 (13.0)
MET amplification			
No	92 (81.4)	70 (77.8)	22 (95.7)
Yes	17 (15.0)	16 (17.8)	1 (4.3)
Unknown	4 (3.5)	4 (4.4)	—
EGFR mutation status			
Exon19del	71 (62.8)	56 (62.2)	15 (65.2)
L858R	28 (24.8)	23 (25.6)	5 (21.7)
Uncommon	5 (4.4)	5 (5.6)	—
Complex	9 (8.0)	6 (6.7)	3 (13.0)

NOTE. Demographic and clinical characteristics of the dual-center cohort consisting of 123 patients with EGFR-mutant NSCLC, categorized by the presence or absence of CTNNB1 mutations. Variables presented include patient demographics (eg, age at diagnosis, sex, smoking history), clinical factors (ECOG performance status, UICC stage, histology, presence of CNS metastasis), and molecular characteristics (PD-L1 expression levels, TP53 mutation status, MET amplification levels, and specific EGFR mutation subtypes). By comparing the CTNNB1-wt group with the CTNNB1-mut group, the table highlights the distribution of these factors and explores their potential implications for treatment outcomes and disease progression within this patient cohort.

Abbreviations: ADC, adenocarcinoma; CTNNB1, catenin beta-1; CTNNB1-mut, CTNNB1-mutant; CTNNB1-wt, CTNNB1-wild-type; ECOG, Eastern Cooperative Oncology Group; EGFR, epidermal growth factor receptor; Ex19del, in-frame deletion within exon 19; NSCLC, non–small cell lung cancer; L858R, leucine to arginine mutation at codon 858; TPS, tumor proportion score; UICC, Union for International Cancer Control.

## References

[1] Herbst RS Morgensztern D Boshoff C The biology and management of non-small cell lung cancer Nature 553 446 454 2018 29364287 10.1038/nature25183

[2] Rosell R Moran T Queralt C et al Screening for epidermal growth factor receptor mutations in lung cancer N Engl J Med 361 958 967 2009 19692684 10.1056/NEJMoa0904554

[3] Janning M Süptitz J Albers-Leischner C et al Treatment outcome of atypical EGFR mutations in the German National Network Genomic Medicine Lung Cancer (nNGM) Ann Oncol 33 602 615 2022 35263633 10.1016/j.annonc.2022.02.225

[4] Soria JC Ohe Y Vansteenkiste J et al Osimertinib in untreated EGFR-mutated advanced non-small-cell lung cancer N Engl J Med 378 113 125 2018 29151359 10.1056/NEJMoa1713137

[5] Christopoulos P Kirchner M Roeper J et al Risk stratification of EGFR(+) lung cancer diagnosed with panel-based next-generation sequencing Lung Cancer 148 105 112 2020 32871455 10.1016/j.lungcan.2020.08.007

[6] Pezzuto F Hofman V Bontoux C et al The significance of co-mutations in EGFR-mutated non-small cell lung cancer: Optimizing the efficacy of targeted therapies? Lung Cancer 181 107249 2023 37244040 10.1016/j.lungcan.2023.107249

[7] Yu F Yu C Li F et al Wnt/β-catenin signaling in cancers and targeted therapies Signal Transduct Target Ther 6 307 2021 34456337 10.1038/s41392-021-00701-5 PMC8403677

[8] Cerami E Gao J Dogrusoz U et al The cBio cancer genomics portal: An open platform for exploring multidimensional cancer genomics data Cancer Discov 2 401 404 2012 22588877 10.1158/2159-8290.CD-12-0095 PMC3956037

[9] Blakely CM Watkins TBK Wu W et al Evolution and clinical impact of co-occurring genetic alterations in advanced-stage EGFR-mutant lung cancers Nat Genet 49 1693 1704 2017 29106415 10.1038/ng.3990 PMC5709185

[10] Yu HA Suzawa K Jordan E et al Concurrent alterations in EGFR-mutant lung cancers associated with resistance to EGFR kinase inhibitors and characterization of MTOR as a mediator of resistance Clin Cancer Res 24 3108 3118 2018 29530932 10.1158/1078-0432.CCR-17-2961 PMC6420806

[11] Arasada RR Shilo K Yamada T et al Notch3-dependent beta-catenin signaling mediates EGFR TKI drug persistence in EGFR mutant NSCLC Nat Commun 9 3198 2018 30097569 10.1038/s41467-018-05626-2 PMC6090531

[12] Fang X Gu P Zhou C et al β-Catenin overexpression is associated with gefitinib resistance in non-small cell lung cancer cells Pulm Pharmacol Ther 28 41 48 2014 23707949 10.1016/j.pupt.2013.05.005

[13] Katagiri H Yonezawa H Shitamura S et al A Wnt/β-catenin signaling inhibitor, IMU1003, suppresses the emergence of osimertinib-resistant colonies from gefitinib-resistant non-small cell lung cancer cells Biochem Biophys Res Commun 645 24 29 2023 36669423 10.1016/j.bbrc.2023.01.018

[14] Togashi Y Hayashi H Terashima M et al Inhibition of beta-Catenin enhances the anticancer effect of irreversible EGFR-TKI in EGFR-mutated non-small-cell lung cancer with a T790M mutation J Thorac Oncol 10 93 101 2015 25384171 10.1097/JTO.0000000000000353

[15] Yi Y Li P Huang Y et al P21-activated kinase 2-mediated beta-catenin signaling promotes cancer stemness and osimertinib resistance in EGFR-mutant non-small-cell lung cancer Oncogene 41 4318 4329 2022 35986102 10.1038/s41388-022-02438-z

[16] Hellyer JA White MN Gardner RM et al Impact of tumor suppressor gene co-mutations on differential response to EGFR TKI therapy in EGFR L858R and exon 19 deletion lung cancer Clin Lung Cancer 23 264 272 2022 34838441 10.1016/j.cllc.2021.09.004

[17] Thomas de Montpréville V Lacroix L Rouleau E et al Non-small cell lung carcinomas with CTNNB1 (beta-catenin) mutations: A clinicopathological study of 26 cases Ann Diagn Pathol 46 151522 2020 32442860 10.1016/j.anndiagpath.2020.151522

[18] Yoo SB Kim YJ Kim H et al Alteration of the E-cadherin/β-catenin complex predicts poor response to epidermal growth factor receptor-tyrosine kinase inhibitor (EGFR-TKI) treatment Ann Surg Oncol 20 S545 S552 2013 suppl 3 23579873 10.1245/s10434-013-2970-1

[19] Kästner A Kron A van den Berg N et al Evaluation of the effectiveness of a nationwide precision medicine program for patients with advanced non-small cell lung cancer in Germany: A historical cohort analysis Lancet Reg Health Eur 36 100788 2024 38034041 10.1016/j.lanepe.2023.100788 PMC10687333

[20] Schemper M Smith TL A note on quantifying follow-up in studies of failure time Control Clin Trials 17 343 346 1996 8889347 10.1016/0197-2456(96)00075-x

[21] Memmott R Gheeya J Wei L et al P3.12D.01 A phase Ib study of osimertinib and tegavivint as first-line therapy in patients with metastatic EGFR-mutant non-small cell lung cancer (NSCLC) J Thorac Oncol 19 S346 2024

[22] Liu R Zhang Y Ding Y et al Characteristics of TGFBR1-EGFR-CTNNB1-CDH1 signaling Axis in Wnt-regulated invasion and migration in lung cancer Cell Transpl 29 963689720969167 2020 10.1177/0963689720969167 PMC7784602 33231090

[23] Pacheco-Pinedo EC Durham AC Stewart KM et al Wnt/β-catenin signaling accelerates mouse lung tumorigenesis by imposing an embryonic distal progenitor phenotype on lung epithelium J Clin Invest 121 1935 1945 2011 21490395 10.1172/JCI44871 PMC3083778

[24] Hewer E Fischer PD Vassella E et al Lymphoid enhancer-binding factor 1 (LEF1) immunostaining as a surrogate for beta-catenin (CTNNB1) mutations J Clin Pathol 2024 10.1136/jcp-2024-209695 PMC12772540 39653501

[25] Canale M Petracci E Delmonte A et al Concomitant TP53 mutation confers worse prognosis in EGFR-mutated non-small cell lung cancer patients treated with TKIs J Clin Med 9 1047 2020 32272775 10.3390/jcm9041047 PMC7230306

[26] Kim Y Lee B Shim JH et al Concurrent genetic alterations predict the progression to target therapy in EGFR-mutated advanced NSCLC J Thorac Oncol 14 193 202 2019 30391576 10.1016/j.jtho.2018.10.150

[27] Roeper J Christopoulos P Falk M et al TP53 co-mutations as an independent prognostic factor in 2nd and further line therapy-EGFR mutated non-small cell lung cancer IV patients treated with osimertinib Transl Lung Cancer Res 11 4 13 2022 35242623 10.21037/tlcr-21-754 PMC8825660

[28] Hamakawa Y Agemi Y Shiba A et al Association of PD-L1 tumor proportion score ≥20% with early resistance to osimertinib in patients with EGFR-mutated NSCLC Cancer Med 12 17788 17797 2023 37548381 10.1002/cam4.6405 PMC10523952

[29] Hsu KH Tseng JS Yang TY et al PD-L1 strong expressions affect the clinical outcomes of osimertinib in treatment naïve advanced EGFR-mutant non-small cell lung cancer patients Sci Rep 12 9753 2022 35697720 10.1038/s41598-022-13102-7 PMC9192769

[30] Papazyan T Denis MG Sagan C et al Impact of PD-L1 expression on the overall survival of caucasian patients with advanced EGFR-mutant NSCLC treated with frontline osimertinib Target Oncol 19 611 621 2024 38825654 10.1007/s11523-024-01072-x

[31] Shiozawa T Numata T Tamura T et al Prognostic implication of PD-L1 expression on osimertinib treatment for EGFR-mutated non-small cell lung cancer Anticancer Res 42 2583 2590 2022 35489768 10.21873/anticanres.15736

[32] Blasi M Kuon J Lüders H et al First-line immunotherapy for lung cancer with MET exon 14 skipping and the relevance of TP53 mutations Eur J Cancer 199 113556 2024 38271745 10.1016/j.ejca.2024.113556

[33] Li M Hou X Chen J et al ALK fusion variant 3a/b, concomitant mutations, and high PD-L1 expression were associated with unfavorable clinical response to second-generation ALK TKIs in patients with advanced ALK-rearranged non-small cell lung cancer (GASTO 1061) Lung Cancer 165 54 62 2022 35091210 10.1016/j.lungcan.2022.01.006

[34] Pai SG Carneiro BA Mota JM et al Wnt/beta-catenin pathway: Modulating anticancer immune response J Hematol Oncol 10 101 2017 28476164 10.1186/s13045-017-0471-6 PMC5420131

[35] Yu HA Kerr K Rolfo CD et al Detection of MET amplification (METamp) in patients with EGFR mutant (m) NSCLC after first-line (1L) osimertinib J Clin Oncol 41 2023 (suppl 16; abstr 9074)

